# Touching body, soul, and spirit? Understanding external applications from integrative medicine: A mixed methods systematic review

**DOI:** 10.3389/fmed.2022.960960

**Published:** 2022-12-22

**Authors:** Inga Mühlenpfordt, Sarah B. Blakeslee, Janina Everding, Holger Cramer, Georg Seifert, Wiebke Stritter

**Affiliations:** ^1^Department of Pediatrics, Division of Oncology and Hematology, Charité – Universitätsmedizin Berlin, Freie Universität Berlin, Humboldt-Universität zu Berlin, Berlin Institute of Health, Berlin, Germany; ^2^Institute of General Practice and Interprofessional Care, University Hospital Tübingen, Tübingen, Germany; ^3^Bosch Health Campus, Stuttgart, Germany; ^4^Department of Pediatrics, Faculty of Medicine, University of São Paulo, São Paulo, Brazil

**Keywords:** external application, integrative medicine, anthroposophic medicine, touch, mixed methods systematic review

## Abstract

**Introduction:**

External applications from anthroposophic medicine (EAAM) are touch-based applications such as rhythmical massages, embrocations, and compresses that serve as components of complementary treatment concepts for various diseases. The aim of this review is to gain an understanding of typical indications and outcomes and to systematically assess the effectiveness and safety of EAAM.

**Materials and methods:**

Medline/PubMed, CINAHL, the Cochrane Library, Embase, and PsycINFO were searched through May 2021 and supplemented by searches in specialized databases and personal requests to experts in the field. Studies and case reports on EAAM in patients, as well as healthy individuals, were included in the qualitative synthesis. Outcome parameters depending on each study were grouped as effect themes and assigned to study clusters using Thematic Analysis for a thematic overview of effect patterns.

**Results:**

Four RCTs, 7 cohort studies, 1 mixed-methods, 1 retrospective, 4 qualitative studies, 3 case series, and 25 case reports on EAAM were identified. The analysis indicated various effects of EAAM on physiological as well as psychological health indicators and patterns of effect development. Study quality was found to be high for only 2 studies, and moderate for 1 study, and all remaining 45 studies showed a moderate or high risk of bias or were not ratable with used rating tools.

**Conclusion:**

The included studies present a wide range of potential indications for EAAM, while showing methodological drawbacks. To determine whether EAAM can be considered an effective treatment option, clinical studies exploring the effect of different EAAM modalities on defined patient groups are recommended for the future.

**Systematic review registration:**

[https://www.crd.york.ac.uk/prospero/display_record.php?RecordID=214030], identifier [CRD42020214030].

## Introduction

Supporting individual patient needs at times goes beyond the limits of conventional medicine. Integrating methods from traditional, complementary, and integrative medicine into treatments may extend therapeutic options and add valuable knowledge on health promotion (1, 2). While biomedical clinical treatments often place great value on the established diagnosis and its specific treatment, integrative treatments offer holistic treatment with a focus on individual health and well-being (3, 4). External applications and massage techniques involving interpersonal attention, touch, and natural substances play a central role in complementary medical systems and are used in various medical conditions (5–9).

### Effect mechanisms of external applications

In general, external applications unfold their effects through several different mechanisms: the effect of physical treatment, the touch of the therapist, a calming environment, and the substance on the skin (10). These factors, as well as warmth and olfaction, have been found to be beneficial for social interaction, emotional state, and relaxation (11). While little is known about the effects of external applications, some of the crucial facets of external applications have been subject to research.

#### Temperature and added substances

The effect of applications with altered temperatures on the organism can be explained by the principle of hormesis, a physiological regulatory reaction to activation by external stimuli or moderate stressors, such as heat or cold (12, 13). These reactions might also result from substances with heating and cooling capacities due to their chemical composition. An example of hormesis on a cellular level was described as the heat shock response, where heat is understood to trigger the organism to activate self-regulatory processes and therefore modulate longevity (14). Natural substances such as essential oils added to external applications are assumed to unfold their aromatherapeutic effects through scent and olfactory receptors on the skin (15). Certain odors may alter endogenous opioid pathways that reduce pain and anxiety and have antibacterial, antifungal, anti-inflammatory, immunomodulatory, and antioxidant effects (16). Positive effects on physiological and psychological parameters have been found with aromatherapy (17, 18), while the effects of aromatherapy on other health indicators have been found to be inconclusive (19). While research on the impact of individual substances on the body is scarce, research within the field of aromatherapy points to challenges in the differentiation of the principle of action of essential oils (20). However, lavender essential oil is presumed to have relaxing and sedative as well as analgesic, anticonvulsive, and neuroprotective capacities (21, 22). Ginger and mustard were found to have warming properties in external hydrotherapeutic treatments (23, 24).

#### Interpersonal touch and massage

Through the complex system of nerve fibers on the skin, the sense of touch is of great importance for haptic and tactile perception and social interaction (25). At the hormonal level, touch can inhibit the release of cortisol, while it can promote the release of dopamine, serotonin, and oxytocin. Oxytocin, in particular, is associated with modulating pain, increasing wound healing, and reducing stress (11, 26–28). Touching the skin with a certain pace and pressure stimulates pressure receptors. This has shown positive affective valence by stimulating especially C-tactile afferents in the skin, leading to increased vagal activity that may mediate the positive effects of touch (29, 30). Psychological effects of touch are shown in improved emotion regulation and attentiveness as well as decreased stress, depression, and anxiety levels (30, 31). The effects of massage therapy have been the subject of many studies, proposing stress relief and effects on symptoms through physiologic and psychological mechanisms, such as improvement in hypothalamus–pituitary–adrenocortical (HPA) axis function, reduction in heart rate, and increase in blood flow as underlying mechanisms (32), resulting in beneficial effects on various health conditions (9).

### External applications in anthroposophic medicine

Anthroposophic medicine (AM) is an integrative medical system developed in Europe around 1900 by Rudolf Steiner and M. D. Ita Wegman. AM is based on the views of the spiritualist movement of Anthroposophy and is intended to be applied in combination with recognized science-based medical methods (33). Aiming at a holistic promotion of health, AM facilitates a multimodal spectrum of methods that are mostly based on experience (34). Medical views in AM are based on the concept of *Four Levels of Formative Forces* in humans consisting of the physical body and its vital processes (*Body*), psychological processes (*Soul*), and the superordinate consciousness (*Spirit*) (33). According to AM, the human organism also consists of interconnected *Threefold Functional Subsystems*: One for sensory perception and consciousness (*Nerve-Sense System*), one for cardiovascular and respiratory function (*Rhythmic System*), and one for muscular activity and volition (*Motor-Metabolic-Limb System*) (34, 35). In AM, etiologies and pathogenesis of diseases are seen as abnormal interactions between these different levels and subsystems and are defined as defective processes of maturation or defective impulses leading the organism to disbalance (34–36). The AM methods spectrum includes the use of medications made from natural products, art and movement therapies, counseling, meditation practice, hydrotherapy, and different massage and nursing techniques in the form of external applications from anthroposophic medicine (EAAM). With the intention to synergize the effects of different therapeutic modalities, AM treatment concepts are often multimodal, individually tailored, and take into account the physical, emotional, mental, spiritual, and social situation of a person (34) and are supposed to address vital processes (37). The therapeutic goals of AM recognize and stimulate self-efficacy by enabling flexible autonomic self-regulation as well as psycho-emotional and spiritual self-regulation (34), a concept raised in a similar manner in *Salutogenesis* where it is described as heterostasis in the body (38, 39).

External applications from anthroposophic medicine are intended to stimulate autonomic self-regulation and salutogenic processes by physically influencing the distribution of warmth in the organism, and therefore, integrating the functions of *Body, Soul, and Spirit* according to AM (40, 41). Applications such as massage including rhythmical massage (RM), rhythmical embrocation (RE), Pressel stream massage (PM), and compress or wrap applications are used. They are performed by trained nurses, physiotherapists, or caregivers. Applications combine interpersonal attention, touch, pressure, and rhythmic movements with temperature with aroma by using tempered water, etheric or fatty oils, essences, tinctures, and ointments on the skin (34). The applications are performed as part of integrative treatment concepts or as single interventions (42, 43). The massages are usually applied 1–2 times a week in units of 30–60 min and cycles of 8–12 treatments, and the duration of the compresses depends on the applied substance (42, 43). All EAAMs are generally followed by a therapeutic resting period of 30 min (42). Table 1 shows an overview of the different types of EAAM.

**TABLE 1:** Types of external applications from anthroposophic medicine.

Application type	Origin	Practice description	Intention
Rhythmical massage	Developed as an independent massage technique in anthroposophic medicine (AM), extending basic movements of classical massage.	Extends basic movements of classical massage by rhythmic, expanding and contracting, circular and lemniscate movements, and movements progressing from the depths to the periphery of the body.	Intended to influence the tissue, as well as the body, soul and spirit through the *Rhythmical System* according to AM (44).
Rhythmical embrocation	Differentiation of specific elements of rhythmical massage.	Using only stroking, embrocation is mainly done in the form of rhythmically performed circles or spirals, perceived more locally on the skin rather than in the deeper tissue.	Intended to influence the body, soul, and spirit through the *Rhythmical System* according to AM (45).
Pressel stream massage	Advanced form of rhythmical massage.	Alternately treating the lower and upper body and/or the left and right side of the body during a treatment sequence. Calves, thighs and the lower back are usually massaged in one session followed by the treatment of the upper back, arms and neck in the next.	Sequences are described to set a stimulating energy flow in motion (46).
Compresses		Application in different body regions using natural substances on the skin. Typical forms are warm wet compresses using *Achillea millefolium* (yarrow) infusion (applied for 30 min), etheric or fatty oils (applied for 30 min and longer), sinapi (mustard) flour (applied for 5, increasing to 12 min), and *Zingiber officinale* (ginger) powder (applied for up to 20 min).	Intended to support self-regulation (42).

#### State of research on external applications from anthroposophic medicine

While literature reviews are available for multimodal application and other treatment modalities of AM (47–51), a systematic review on EAAM has not yet been published and is necessary for establishing the safety and evaluating the effectiveness of the method.

### Aims of the review

Given the research gap regarding EAAM, the overall aim of this mixed methods systematic review is to clarify existing applications and indications and to assess the effects and safety of EAAM. This aim has three specific outcomes: the analysis will describe typical indications for EAAM, typical outcomes will be summarized, and the effectiveness and safety will be systematically assessed.

## Materials and methods

The review was planned following the principles of a mixed methods systematic review (52) and registered in the *International Prospective Register of Systematic Reviews* (PROSPERO) (Registration ID: CRD42020214030). The study selection and assessment were conducted in accordance with *Preferred Reporting Items for Systematic Reviews and Meta-Analyses* (PRISMA) guidelines (53) and following the recommendations of the *Cochrane Collaboration* (54). Data analysis and synthesis were conducted qualitatively.

### Eligibility criteria

For the description of eligibility criteria, refer to Table 2. Only isolated EAAM interventions were included; multimodal treatments, for instance as part of a wider AM regimen, were excluded. Hydrotherapy interventions were excluded since they do not necessarily involve interpersonal touch. Interventions that used EAAM-like applications but not specified as applications of AM were excluded as well.

**TABLE 2:** Eligibility criteria.

Criteria	Inclusion
Study types	Quantitative studies, e.g., randomized controlled trials (RCTs); quasi-experimental studies; qualitative studies; case studies and reports.
Publication	Peer-reviewed publications, gray literature records, extensive abstracts.
Language	English or German.
Participants	No restrictions on the types of participants, symptoms, and indications.
Interventions	Interventions involving direct interpersonal touch, including rhythmical massage, rhythmical embrocation, Pressel stream massage, compresses and wraps. Studies could test external applications from anthroposophic medicine (EAAM) either as an adjuvant to other modalities/therapies or as a single therapeutic approach. Only studies examining the effects of EAAM in isolation from other treatment modalities were included.
Outcome measures	Any health indicators, such as somatic and psychological parameters.

### Search strategy

The literature search was built around search terms for “anthroposophic medicine” and “External Applications.” The following electronic databases were searched from their inception through 31 May 2021: Medline/PubMed, CINAHL, the Cochrane Library, Embase, and PsycINFO. The Anthromedics Merkurstab archive^1^, CAMbase^2^, CAM-Quest^3^, ResearchGate^4^, and the System for Information on Grey Literature in Europe^5^ for inclusion of gray literature. The search strategy was adapted for each database as necessary and conducted in English and/or German, depending on the database. Additionally, 104 international experts and professional institutions of AM were asked to contribute articles of relevance by direct contact *via* e-mail from 15 April 2019 and submissions were accepted until 31 May 2021. The complete search strategy for each database is shown in Table 3. Finally, reference lists of identified original articles were searched manually.

**TABLE 3:** Search strategy.

Source type	Source	Terms/Strategy
Electronic data bases	Medline/PubMed	anthroposoph*[All Fields] AND (extern*[Title/Abstract] OR rhythmic*[Title/Abstract] OR massage[Title/Abstract] OR embrocation[Title/Abstract] OR wash*[Title/Abstract] OR compress[Title/Abstract] OR pack[Title/Abstract] OR poultice[Title/Abstract] OR wrap[Title/Abstract] OR essence[Title/Abstract] OR liniment[Title/Abstract] OR ointment[Title/Abstract] OR oil[Title/Abstract] OR tincture[Title/Abstract])
	CINAHL	*FULLTEXT:* anthroposoph* AND *ABSTRACT:* (extern* OR rhythmic* OR massage OR embrocation OR wash* OR compress OR pack OR poultice OR wrap OR essence OR liniment OR ointment OR oil OR tincture)
	Cochrane Library	anthroposoph* AND (extern* OR rhythmic* OR massage OR embrocation OR wash* OR compress OR pack OR poultice OR wrap OR essence OR liniment OR ointment OR oil OR tincture)
	Embase	anthroposoph* AND (extern* OR rhythmic* OR massage OR embrocation OR wash* OR poultice OR wrap OR pack OR compress OR essence OR liniment OR ointment OR oil OR tincture)
	PsycINFO	*FULLTEXT:* anthroposoph* AND *ABSTRACT:* (extern* OR rhythmic* OR massage OR embrocation OR wash* OR compress OR pack OR poultice OR wrap OR essence OR liniment OR ointment OR oil OR tincture)
Alternative electronic data bases	Anthromedics Merkurstab data base	*MERKURSTAB HAUPTARTIKEL: TITEL:* extern* OR rhythm* OR massage OR embrocation OR wash* OR compress OR pack OR poultice OR wrap OR essence OR liniment OR ointment OR oil OR tinctur* OR äußer* OR äusser* OR aeußer* OR aeusser* OR Einreibung OR Wasch* OR Wickel OR Auflage OR Salbe OR Öl OR Oel
	CAMbase	anthroposoph* AND (extern* OR rhythm* OR massage OR embrocation OR wash* OR compress OR pack OR poultice OR wrap OR essence OR liniment OR ointment OR oil OR tinctur* OR äußer* OR äusser* OR aeußer* OR aeusser* OR Einreibung OR Wasch* OR Wickel OR Auflage OR Salbe OR Öl OR Oel)
	CAM-Quest	CATEGORY: “Anthroposophische Medizin”: rhythmic; massage; embrocation; compress; pack; poultice; wrap; wash; Rhythmische; Massage; Einreibung; Wickel; Auflage; Waschung
	Open gray	anthroposophy; anthroposophic; Anthroposophie
	ResearchGate	*PUBLICATIONS: ALL TYPES:* anthroposoph* AND (massage OR embrocation OR wash* OR compress OR pack OR poultice OR wrap OR Einreibung OR Wickel OR Auflage OR Wasch*)
Personal inquiry	Experts and institutions	E-mail inquiry to a total of 104 international experts and institutions of anthroposophic medicine and nursing

### Qualitative analysis of studies

Using a *Thematic Analysis* approach to identify, analyze, and report patterns (themes) within the data, an inductive text analysis of the included records was carried out with MAXQDA2020 (Release 20.4.1) (55). First, the result sections of the included studies were coded according to the described effects on outcomes. Then, key themes regarding the treatment effects were generated based on these codes and summarized in a brief description of the subthemes separately for massage and compress studies. Subsequently, significant effects (*p* ≤ 0.05) of massages and compresses on participants were summarized for each of the generated themes.

### Quality assessment of the individual studies

Since studies of different methodologies were eligible for inclusion the assessment of study quality was undertaken in 3 steps. First, we sorted the studies by the level of evidence. For the detailed quality assessment, the recommended tool for risk of bias assessment of controlled trials in systematic reviews *Cochrane Risk of Bias Tool* (RoB) (56) was used and supplemented by the *Quality Assessment Tool for quantitative studies* (QA-Tool) by the Effective Public Health Practice Project (57). This quality assessment provided insight into the available study types and appropriate classification of studies according to their methodology.

All assessments were conducted by IM. Half of the QA-Tool assessments as well as half of the RoB assessments were also performed by JE independently. The remaining assessments were reviewed by JE. Conflicts of rating were discussed until a consensus was reached.

#### Level of evidence assessment

We first performed a Level of Evidence (LoE) assessment for all included studies, using an adapted version of the *Evidence-based Nursing Care Guidelines Scheme* (58), p. 7; as a heuristic framework assigning LoE by 7 levels.

#### Study quality assessment

For the quality assessment of the quantitative studies, we used the QA-Tool. The tool assesses study quality according to a strong, moderate, or weak rating quality along with the following domains: selection bias, study design, confounders, blinding, data collection methods, withdrawals and dropouts, intervention integrity, and analyses (57). Studies with no weak component rating were rated as having overall strong quality, studies with one weak component rating were rated as having moderate quality, and studies with two or more weak ratings were rated as having weak quality.

#### Risk of bias assessment

The RoB tool assesses the risk of bias in the domains of selection bias, performance bias, attrition bias, reporting bias, and detection bias using 8 criteria (56). Each criterion in the quantitative studies was rated as low risk, unclear risk, or high risk of bias. To provide comparability with the QA-tool, we supplemented the RoB-rating with an assessment of the overall risk of bias. Studies that met at least six of the criteria were rated as having an overall low risk of bias, studies that met four to five criteria were rated as having a moderate risk of bias, and studies that met zero to three criteria were rated as having a high risk of bias.

## Results

### Literature search

The literature search revealed a total of 665 records, resulting in 390 non-duplicate records of which 238 were excluded because they did not report on EAAM studies in English or German. Out of 144 full-texts assessed for eligibility, 99 articles were excluded because they were theoretical descriptions or instructions of EAAM (58 records), were double publications on the same study (25 records), reported epidemiological or ethnographic data (7 records), were not studied on EAAM (6 records), or were study protocols without results (3 records).

**FIGURE 1:**
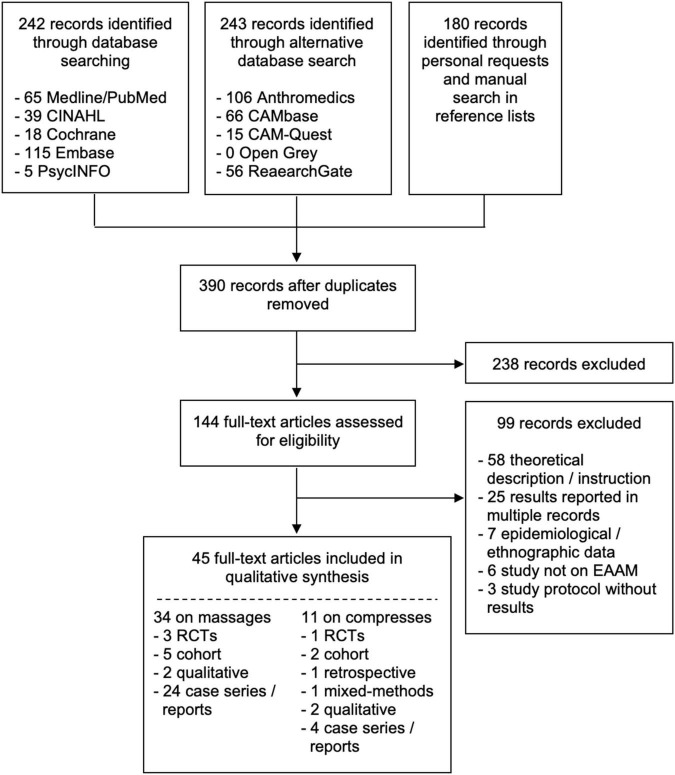
Flowchart of the results of the literature search.

Finally, 45 studies were included, out of these 34 studies were on massage interventions and 11 were on compresses interventions. On massages, there were 3 RCTs, 5 cohort studies, 2 qualitative studies, and 24 case reports or series. On compresses, there were 1 RCT, 1 mixed methods study, 2 cohort studies, 1 retrospective study, 2 qualitative studies, and 4 case reports or series. The flowchart of the results of the literature search is depicted in Figure 1.

### Publication characteristics

Of the 45 studies, 18 were published in peer-reviewed journals (59–76), and 27 studies were records of gray literature (77–103). Fifteen studies were only available in the form of an abstract (77, 80, 82, 85, 87, 88, 90–95, 97, 101, 102).

**FIGURE 2:**
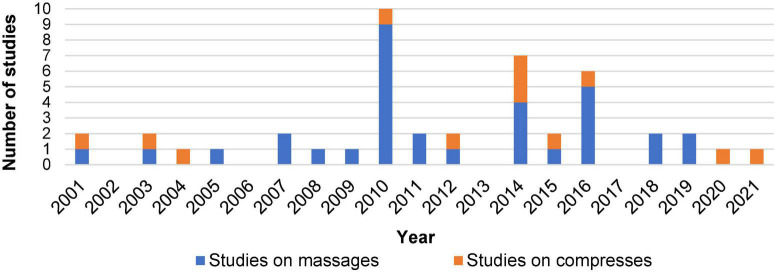
The number of studies on EAAM in a given year reported in absolute terms. Studies exploring a mixed intervention of massages and other application types were counted as massage studies.

The first included study was published in 2001, in fact as the second edition of a study originally published in 1992 (98). The number of studies published each year reached a peak between the years 2010 and 2016 (Figure 2).

### Study characteristics

Characteristics of the study methodologies, samples, interventions, outcome assessments, and results are shown in Tables 4–9. The studies in the result tables are sorted by their assigned LoE and grouped in tables per studies on massages and studies on compresses.

**TABLE 4:** Summary of quantitative studies on massages.

Reference	Type of study	Participants: *N*, diagnosis, age	Intervention: Modality, substance, body region, number/time span	Main outcome assessment	Main results: Improvements of outcome parameters	Level of evidence, quality, risk of bias
Kanitz et al. (70)	RCT, 3 groups, single-blinded	*N* = 101, healthy adults, 25.2 ± 4.7 years	TSST followed by 1× treatment in individualized body regions, 3 groups: (1) RA: RM with aroma oil with peat and lavender extracts, (2) RM with jojoba oil, (3) SM with jojoba oil (control)	(A) Well-being: MDBF, Bf-S, assessment BT, AT (B) Somatic complaints: B-L, VAS; assessment BT, AT (C) Salivary cortisol; assessment at 5 points (D) Open questions; assessment AT	BG and AT vs. BT (A) ns (B) ns (C) ns (D) Description of application as relaxing after treatment in the groups: (1) 100% (2) 82% (3) 42%	LoE: II QA: Strong RoB: Moderate risk
Seifert et al. (69)	RCT, 3 groups, single-blinded	*N* = 44, female healthy adults, 26.2 ± 4.71 years [Selection of the sample of Kanitz et al. (70)]	TSST followed by 1× treatment in individualized body regions, 3 groups: (1) RA: RM with aroma oil with peat and lavender extracts, (2) RM with jojoba oil, (3) SM with jojoba oil (control)	Autonomic regulation: HRV parameters of ECG; 24-h assessment of 12 parameters: BT (T1), after massage (T2), after 12 h (T3), after 24 h (T4)	BG: Most of the HRV parameters: (1) vs. (2): *p* < 0.01 (1) vs. (3): *p* < 0.01 (2) vs. (3): *p* < 0.01 BG after 24 h: Most of the HRV parameters: (1) and (2) vs. (3): *p* < 0.01	LoE: II QA: Strong RoB: Moderate risk
Vagedes et al. (73)	RCT, 3 groups, not blinded	*N* = 60, female with dysmenorrhea, 29.7 ± 8.0 years	Treatment over 3 months, 3 groups: (1) RM, substance/region unspecified, 12×, weekly (2) HRV-Biofeedback: Daily, training/booster sessions every 4 weeks (3) Waiting control, TAU	(A) Pain intensity during menstruation: NRS (B) QoL: SF-12 (C) HRV: 24-h ECG (D) Use of analgesics; assessment BT and AT	(A) BG: (1) vs. (3): *p* < 0.01, *d* = −0.80, (1) vs. (2): ns, (2) vs. (3): ns (B) BG: ns, AT vs. BT in (1): *d* = 0.60, (C) BG: ns, AT vs. BT in (1): ns (D) (1) Reduction from 70% BT to 60% AT	LoE: II QA: Weak RoB: Moderate risk
Hamre et al. (65)	Cohort study, 1 group, not blinded	*N* = 85, chronic diseases (musculoskeletal, mental etc.), 35.7 ± 19.3 years	RM, substance/region unspecified, 12× (median) within 84 days (median)	(A) Disease score (B) Symptom score (C) QoL: SF-36 scales (D) Therapy ratings: Overall, satisfaction, effectiveness (0–10) (E) AE; assessment BT and after 3, 6, 12, 18, 24, and 48 months	12 months after start vs. BT: (A) *p* < 0.001, *d* = 1.45 (B) *p* < 0.001, *d* = 1.14 (C) Physical Health: *p* < 0.001, *d* = 0.57, Mental Health: *p* = 0.001, *d* = 0.63 (D) Overall rating: 7.50 ± 2.34, satisfaction: 8.18 ± 2.08, Effectiveness: Positive rating in 83% patients, 77% physicians; continued good ratings of satisfaction/effectiveness in follow-ups (E) In 4 cases: Cardiac palpitations, arterial hypotension, pain and vertigo, symptom aggravation	LoE: IV QA: Weak RoB: High risk
Wälchli et al. (67)	Cohort study, 1 group, not blinded	*N* = 59, any indication, 49.4 ± 12.5 years	RM with individualized oils/ointments, in individualized body regions, 9× (average), 1–2× per week within 3 months	(A) Disease score (B) Symptom score (C) QoL: SF-36 (D) Goal attainment: GAS (E) AE; assessment BT, AT, 6 months after first session	AT vs. BT/after 6 months vs. BT: (A) *p* < 0.001/ < 0.001 (B) *p* < 0.001/ < 0.001 (C) Physical Health: *p* = 0.005/ = 0.008, Mental Health: *p* < 0.001/*p* < 0.001 (D) *p* < 0.001/*p* < 0.001 (E) None	LoE: IV QA: Weak RoB: High risk
Wälchli et al. (66)	Cohort study, 1 group, not blinded	*N* = 13, any indication, 54.8 ± 3.8 years, IRI: *n* = 9, ECG: *n* = 11 [Selection of the sample of Wälchli et al. (67)]	RM with individualized oils/ointments, in individualized body regions, 10× (average), 1–2 × per week within 3 months	(A) Surface temperature: IRI of dorsal region; assessment in waiting periods BT and AT (B) Autonomic regulation: HRV parameters of ECG; assessment of 6 parameters in waiting periods BT and AT, and after therapeutic rest	AT vs. BT: (A) *p* < 0.001 (B) *p* = 0.0105/0.0001/0.0001/0.0353/0.3909/0.0001 (different HRV parameters), further regulation of HRV during the therapeutic rest	LoE: IV QA: Weak RoB: High risk
Ostermann et al. (64)	Cohort study, 1 group, not blinded	*N* = 100, chronic pain, 47.3 ± 13.9 years	RE with aroma oil with peat and lavender extracts, in individualized body regions, 3× within 24 days	(A) Mental state: Bf-S mood scale (B) Sensory pain: MPQ (C) Affective pain: MPQ (D) Pain intensity: VAS before-after RE (E) Associated medication (F) AE; assessment BT, after each treatment, AT	AT vs. BT: (A) *p* < 0.001, *d* = 0.81 (B) *p* < 0.001, *d* = 0.55 (C) *p* < 0.001, *d* = 0.85 (D) *p* < 0.001, consistent reduction after each treatment (E) No change reported (F) None	LoE: IV QA: Weak RoB: High risk
Vieira et al. (94) (gray, abstract)	Cohort study, 1 group, not blinded	*N* = 27, unspecified geriatric patients	Threefold External Therapy: Body sliding/organ rubbing/foot baths/compresses, substances unspecified, individualized treatment, 12 weekly sessions	(A) Cognitive function: MMSE (B) Incidental memory: FT (C) Immediate memory: FT (D) Evocation: FT (E) Visual perception: FT (F) Nomination: FT (G) Depression: DSM-IV (H) QoL: WHOQOL-bref; assessment BT and AT	AT vs. BT: (A) *p* = 0.008 (B) *p* = 0.003 (C) *p* = 0.006 (D) *p* = 0.001 (E) ns (F) ns (G) *p* = 0.002 (H) Physical: *p* = 0.05, psychological: *p* = 0.007, social: *p* = 0.048, environment: *p* = 0.02	LoE: IV QA: Weak RoB: High risk

Abstract, only abstract of study available; AE, adverse effects; AM, anthroposophic medicine; AT, after treatment; B-L, 24-item list of somatic complaints; Bf-S, Zerssens Adjective Mood Scale for assessment of mental state; BG, between groups; BT, before treatment; *d*, Cohen’s *d* (reported when available); DSM-IV, Diagnostic and Statistical Manual of Mental Disorders edition IV; ECG, electrocardiogram; GAS, Goal Attainment Scale; FT, figures test. HRV-BF, Heart Rate Variability biofeedback; IRI, infrared imaging; LoE, level of evidence based on the evidence-based nursing care guidelines scheme [Ackley et al. (58), p. 7]—rating on level I to VII (see text); MDBF, multidimensional questionnaire on mental state; MMSE, mini-mental state exam; MPQ, McGill Pain Questionnaire; ns, not significant; NRS, Numeric Rating Scale; *p*, *p*-value (reported when available); PPS, Pain Perception Scale; QA, quality assessment according to the Effective Public Health Practice Project scheme (57)—rating 8 criteria and global rating as strong, moderate, or weak (see text); RA, rhythmical massage with aroma oil; RE, rhythmical embrocation; RM, rhythmical massage; RoB, risk of bias assessment according to the Cochrane Collaboration’s tool for assessing risk of bias in randomized trials (56)—rating on 7 domains and global rating as low, moderate, or high risk of bias (see text); QoL, quality of life; SF-36, Health-Related Quality-of-Life questionnaire short form; SF-12, health survey short form of SF-36; SM, Sham massage; TAU, treatment as usual; TSST, Trier Social Stress Test; VAS, Visual Analog Scale; WHOQOL-bref, World Health Organization Quality of Life questionnaire short form.

**TABLE 5:** Summary of quantitative studies on compresses.

Reference	Type of study	Participants: *N*, diagnosis, age	Intervention: Modality, substance, body region, number/time span	Main outcome assessment	Main results: Improvements of outcome parameters	Level of evidence, quality, risk of bias
Ghadjar et al. (72)	RCT pilot, 2 groups, not blinded	*N* = 24, cancer, during palliative RT, 58.5 (median), 34–83 years	2 groups (1) Yarrow liver compress, 10.5×, daily (average) (2) Waiting control, TAU	(A) Fatigue: MFI-20 Subscale (B) Psychological distress: Distress thermometer (C) QoL: QLQ-C30 (D) Symptoms: VAS; assessment BT, after 1 week, AT	BG: (A) General Fatigue scale: ns (*p* = 0.13), but clinically relevant difference of 2 points, Reduced Motivation scale: *p* = 0.035 (B) ns (C) ns (D) Fatigue: *p* = 0.015, tension: *p* = 0.044, lack of drive: *p* = 0.028	LoE: IV QA: Weak RoB: High risk
Stritter et al. (71)	Qualitative part of a mixed methods study: Case cross-over, 1 group, single-blinded (qualitative results: see Table 7)	*N* = 30, healthy adults, 27 ± 4.44 years	4 different chest compresses over 4 weeks in 1 group: (1) Dry (2) Hot water (3) Hot with ginger powder (4) Hot with mustard flour	(A) Somatic complaints: B-L (B) Calmness: MDBF (C) Mood: MDBF (D) Experience: RS (E) AE; assessment BT, after each application	AT vs. BT per each treatment: (A) All compresses: ns (B) All compresses: *p* < 0.05 (C) (1) *p* = 0.03, (2) *p* = 0.01, (3) *p* < 0.001, (4) ns (D) (1) ns, (2) ns, (3) Relaxation: *p* < 0.001, emotional balance: *p* = 0.03, deep breathing: *p* = 0.03, slow breathing: *p* < 0.001, warm hands: *p* = 0.03, warm feet: *p* = 0.00, (4) ns (E) None	LoE: IV QA: Moderate RoB: High risk
Klich-Heartt (100) (gray)	Cohort study, 1 group, not blinded	*N* = 10, fever, 20–74 years	Calf compress with lemon aroma oil, 1×	(A) Temperature: Clinical thermometer (B) Subjective perception of headaches: RS (C) Pulse, respiratory rate, blood pressure; assessment BT, 30 min. AT, and (A) also 60 min. AT	AT vs. BT: (A) *p* = 0.027, BT vs. 60 min. AT: *p* = 0.006 (B) *p* = 0.01 (C) ns	LoE: IV QA: Weak RoB: High risk
Therkleson (62)	Cohort study, 2 treatment groups, not blinded	*N* = 20, chronic osteoarthritis, 64 (mean) years	Ginger compresses, 2 treatment groups, both 1-week daily ginger patch/compress applied to the midlumbar region: (1) Manually prepared ginger compress (2) Ginger patch; additional 24 weeks of self-treatment at home with the patch as required	(A) Health assessment: Arthritis MHAQ/HAQ-II (B) Health satisfaction: RS (C) Use of analgesics; assessment for 21-days: daily/1 week BT, 12 h AT, 1 week AT, 4-weekly for 24 weeks	BG: ns, both groups reported together in percentages of patients, (A) Improvement in mean scores 1 week AT vs. BT: Pain: 48%, fatigue: 49%, GEO: 40%, functional status: 31%; improvement in all scores for all participants in all domains over following 24 weeks of self-treatment (B) BT: 80% dissatisfied, 7 days AT: 70% satisfied, 24 weeks AT: 82% satisfied (C) BT: 70%, 4 weeks AT: 15%	LoE: IV QA: Weak RoB: High risk
Simoes-Wüst et al. (68)	Retrospective descriptive study, 1 group, not blinded	*N* = 221, any indication, 49,6 ± 19,2 years	Various compress types, patients treated with at least one compress during their hospital stay	State of health: 13 items; one-time assessment	Improvement in state of health attributed to the compresses: 70%, considerable recovery: 85%, considerable success of compresses: 76%	LoE: VI QA: Weak RoB: High risk

AT, after treatment; B-L, list of somatic complaints; BT, before treatment; distress thermometer, German version of the distress thermometer of the National Comprehensive Cancer Network; GEO, global effect of osteoarthritis; HAQ-II, Health Assessment Questionnaire II; LoE, level of evidence based on the evidence-based nursing care guidelines scheme [Ackley et al. (58), p. 7]—rating on Level I to VII (see text); MDBF, Questionnaire on mental state; MFI-20, multidimensional fatigue inventory; MHAQ, Modified Health Assessment Questionnaire; ns, not significant; *p*, *p*-value (reported when available); QA, quality assessment according to the Effective Public Health Practice Project scheme (57)—rating 8 criteria and global rating as strong, moderate, or weak (see text); QLQ-C30, quality of life questionnaire of the European Organization for Research and Treatment of Cancer; QoL, quality of life; RoB, risk of bias assessment according to the Cochrane Collaboration’s tool for assessing risk of bias in randomized trials (56)—rating on 7 domains and global rating as low, moderate, or high risk of bias (see text); RS, Subjective Rating Scale; RT, radiation therapy; TAU, treatment as usual; VAS, Visual Analog Scale.

**TABLE 6:** Summary of qualitative studies on massages.

Reference	Type of study	Participants: *N*, diagnosis, age	Intervention: Modality, substance, body region, number/time span	Main outcome assessment	Main results: Improvements of outcome parameters
Berger et al. (59)	Qualitative study, selection of the sample of Vagedes et al. (73)	*N* = 13, female, dysmenorrhea, 16–46 years [those treated with RM, other results see Vagedes et al. (73)]	RM, substance/region unspecified, 12×, weekly over 3 months	Semi-structured interviews, drawings (*n* = 6), questionnaire 1 year after intervention (*n* = 7)	Perception of RM as soft and overall beneficial, feeling different to conventional massage, influence on the whole body; improved pain management, calming, relaxing, increased self-awareness, cause for a process of transformation; reactions depend on the emotional state and readiness to resonate with the therapeutic process
Bertram et al. (60)	Qualitative study, phenomenology	*N* = 13 nursing experts on RE	RE (theoretical)	Semi-structured interviews	Psychosomatic reactions to RE rely on changes in physical parameters and changes in vegetative, mental and spiritual dimensions; Key patterns of patient reaction process: Being uncaged à re-identifying à being empowered

Gray, study from gray literature; RE, rhythmical embrocation; RM, rhythmical massage.

**TABLE 7:** Summary of qualitative studies on compresses.

Reference	Type of study	Participants: *N*, diagnosis, age	Intervention: Modality, substance, body region, number/time span	Main outcome assessment	Main results: Improvements of outcome parameters
Stritter et al. (71)	Qualitative part of a mixed methods study: Case cross-over, 1 group, single-blinded (Quantitative results: see Table 5)	*N* = 15, healthy adults, 27 ± 4.44 years	4 different chest compresses over 4 weeks in 1 group: (1) Dry (2) Hot water (3) Hot with ginger powder (4) Hot with mustard flour	Interviews after each application, follow-up interview	Different onset of relaxation after all 4 applications and resting periods, different qualities of warmth through the ingredients ginger and mustard; (3) Ginger: Most warming and relaxing, instant relaxation, spread of warmth into the whole body, feeling alert and well rested afterward; (4) Mustard: Initial tension, perception of heat mostly in compress area, burning, sudden and strong onset of relaxation in resting period after application
Therkleson and Sherwood (61)	Qualitative study, phenomenology	*N* = 7, various indications, 21–54 years	Ginger kidney-compress, 1×	Semi-structured interview	4 key themes: - Warmth in the body, increasing in intensity and radiating outward - Stimulation of internal activity within the body - Changes in thought-life, sensory perception, and body tension - Centeredness within oneself and greater sense of personal boundary in relation to the world
Therkleson (74)	Qualitative study, phenomenology	*N* = 10, osteoarthritis, >45 years	Ginger kidney-compress on 7 consecutive days	Daily diaries, drawings, personal/phone follow-up interviews	Unique qualities of heat, stimulation, anti-inflammation, and analgesia; 7 key themes: - Meditative-like stillness, relaxation of thoughts - Constant penetrating warmth in the body - Positive change in outlook - Increased energy and interest in the world - Deeply relaxed state with a gradual shift in pain and increased interest in others - Increased suppleness within the body - More comfortable, flexible joint mobility

**TABLE 8:** Summary of case reports on massages.

Reference	Type of study	Participant: Age/gender, diagnosis	Intervention: Modality, substance, body region, number/time span	Main outcome assessment	Main results: Improvements of outcome parameters
Gierse (97) (gray, abstract)	Prospective case study	78/female, after surgical treatment of a humerus fracture	RM, substance/region/number unspecified	Observation, interviews, schematic progress documentation	Warming sensation, deepened breath, improvement of pain, hypoesthesia and mobility, improvement of mental state
Göbels and Allmer (99) (gray)	Prospective case study	39/female, mammary carcinoma, after bilateral mastectomy	RM, substance/region unspecified, 7× over 4 weeks	Observation, interviews, schematic progress documentation, guided diary for self-observation, SF-36; assessment each time	Positive effects on warmth regulation, body experience, emotional well-being, improved emotional well-being
Klocker (101) (gray, abstract)	Prospective case study	Unspecified/male, burnout syndrome	RM, different regions, substance/number unspecified, in 2 cycles	Observation, interviews, schematic progress documentation	Muscle relaxation, warming sensation, harmonizing and deepening the breath, improved self-observation/self-awareness and self-reflection, improved drive
Kögler (102) (gray, abstract)	Prospective case study	47/female, lumbar syndrome	RM, substance unspecified, different regions, 14× in 2 cycles	Observation, interviews, schematic progress documentation	Reduction of pain intensity and frequency of pain attacks, muscle relaxation, warming sensation, harmonizing and deepening the breath, improved decisiveness
Radünz (82) (gray, abstract)	Prospective case study	41/male, obstructive sleep apnea	RM, substance/region unspecified, 10×	Observation, interviews, schematic progress documentation, SF-36; assessment each time	Reduced vertigo, increased appetite, improved sleep, strengthening feeling, less fatigue and improved concentration, anxiety and panic states become increasingly weaker, increase in libido, improved self-awareness
Schober (87) (gray, abstract)	Prospective case study	17/male, spastic tetraparesis	RE, substance/region unspecified, 14× in 7 weeks	Observation, interviews, schematic progress documentation	Warming sensation in the whole body, improved mobility, loosening feeling, improved gross and fine motor skills, vitalizing feeling
Schwarz (88) (gray, abstract)	Prospective case study	64/male, Morbus Parkinson	RM, substance unspecified, different regions, 11× in 11 weeks	Observation, interviews, schematic progress documentation, medical findings of the general practitioner, diary	Increased flexibility in facial expression, deepened breathing, improved balance and upright posture, improved sense of smell, improved sleep and mood
Schwinger (90) (gray, abstract)	Prospective case study	61/female, burnout syndrome	RM, substance/region unspecified, 21× weekly in 3 cycles over 6 months	Observation, interviews, schematic progress documentation, SF-36, assessment each time	Improved self-awareness, mood, well-being, and sleep, vitalizing and relaxing effect
Seedheeyan et al. (91) (gray, abstract)	Prospective case study	50/female, hypoxic brain injury, hypersensitivity to sensory stimuli, pain	RM, substance/region/number unspecified	Pain assessment tools: Body map to indicate location, Wong-Baker Faces Pain Rating Scale, response to qualitative pain description words	Reduced levels of hypersensitivity, anxiety and confusion, improved self-awareness: perception of and ability to describe pain
Uhlenhoff (93) (gray, abstract)	Prospective case study	11/male, on the autism spectrum	RM, substance/region unspecified, 14× biweekly in 7 weeks	Observation, interviews, schematic progress documentation, SF-36	Warming sensation, deepened breath, increased tolerance for touch; AE: discomfort caused by high sensitivity to touch
Amman Albertin (77) (gray, abstract)	Retrospective case report	68/female, asthma bronchiale, hypertension, bilateral osteoarthritis	RM, substance/region/number unspecified	Use of clinical and personal records of the patient	Improvements in asthma symptoms, withdrawal of pharmaceutical therapy, decrease in blood pressure
Börner (78) (gray)	Retrospective case report	6/male, restlessness, sleeping problems, social anxiety, and conflicts	RM, substance/region unspecified, weekly for 6 months	Observation, interviews with parents, schematic progress documentation	Relaxation, improved sleep, improved social interaction
Maier-Schnorr (103) (gray)	Retrospective case report	38/female, migraines, vomiting, nausea, restless, mood swings,	RM of the lower back, hips, abdomen, arms, substance/number unspecified	Observation, interviews	Reduction of vomiting, improvement in restlessness, mood swings, increased attention on avoiding personal overexertion
Meyer (79)(gray)	Retrospective case report	male, pelvic obliquity, scoliosis, muscle tension in the lumbar spine region	RM of the lower back, substance unspecified, biweekly for 3 months, occasional follow-up in following months	Observation, interviews	Improved perception of and strength in the back, ability to lift heavy loads and to work physically without triggering pain or cramps, feeling of well-being and possibility to become active
Vajnai et al. (92) (gray, abstract)	Retrospective case series	59/male, mid-cerebral artery stroke, pain, and spasticity after surgery	RM, substance/region/number unspecified	Observation, interviews	Improved pain management and spasticity management
Weidtke (96) (gray)	Retrospective case series	41/female, operated mammary carcinoma	RM, 3× per week, substance/region/timeframe unspecified	Self-report	Warming sensation, relief of congestion after 1 or 2 days, relieving sensation, relaxing/flowing/healing feeling, feeling of comfort during the after-rest
		68/female, metastasized mammary carcinoma	RM, substance/region/number unspecified	Self-report	Deepened breath, deep relaxation, vitalizing, harmonizing, strengthening, releasing and re-identifying feeling
		50/female, sigmoid carcinoma	RM with various oils (melissa, aurum, lavendula, sloe), region unspecified, 3×	Observation, interviews, schematic progress documentation	Increased comfort in the body, strengthening feeling
Praxl (80) (gray, abstract)	Prospective case study	Not specified/female, hereditary motor sensory neuropathy	RE, substance/region unspecified, 10× in 7 weeks	Observation, interviews, schematic progress documentation, diary	Warming sensation in the legs, improved sensibility in the legs and walk, improvement in sleep and mood, deep relaxation during RE
Reisinger (85) (gray, abstract)	Prospective case study	55/female, depression, multiple abdominal complaints	RE, substance/region unspecified, 7× in 7 weeks	Observation, interviews, schematic progress documentation	Improvement of bloating and abdominal complaints, warming sensation in the whole body, improved wellbeing
Roggatz (84) (gray)	Prospective case study	79/female, sleep disturbance	RE with aroma oil with peat and lavender extracts, on the feet and calves, in the evening for 4–6 weeks	Observation, interviews	RE feel beneficial, pleasant heaviness in the body, light feeling in the feet, relaxation of the body: slower breathing, calmer pulse, relaxed muscles, enhanced warming of the skin after each time; first not calming, but calming down and falling asleep the after rest; after multiple treatments: regularly fast asleep after the RE, less waking in the night, discontinuation of sedative, more relaxed and better sleep
Voit (95) (gray, abstract)	Prospective case study	23/female, exam anxiety	RE, substance/region unspecified, 7× in 7 weeks	Observation, interviews, schematic progress documentation, SF-36, assessment each time	Warming sensation, regulating effect on anxiety and well-being, relaxing and releasing, improved self-awareness and confidence
Pressel (81) (gray)	Retrospective case series	21/female, neck tension, sleep disturbances, amenorrhea	PM in classic regimen, substance unspecified, 6×	Observation, interviews	Improvement of neck tension, return of menorrhea and stable cycle, improved sleep
		34/female, sleep disturbances, depression	PM calf and back massages, substance unspecified, 40×		AE: Headaches, hemorrhage on the calf with accompanying swelling and sensitivity throughout the leg, tiredness, improved sleep
		53/female, chronic fatigue, sleep disturbance	PM calf and back massages, substance unspecified, 10×		Initiation of menstrual bleeding, release of headache, feeling of release and inner alignment, increased well-being
		56/female, recurrent cystitis and appendicitis	PM calf massage, substance unspecified, 1×		Following day after massage: severe pain in the lower abdomen and discharge of blood coagulum, no further bleeding in the following 4 years; AE: Feeling of depression in the evening after massage
Robert (83) (gray)	Retrospective case report	65/female, chronic pain syndrome, chronic depression	PM in classic regimen, substance/number unspecified, weekly sessions	Observation, interviews	Improvement of mobility, sensation of self-healing powers and revitalization, feeling of well-being and new drive
Therkleson and Stronach (75)	Prospective case study	82/female, psychological trauma/characteris-tics diagnosed as Broken Heart Syndrome	RM with aroma oil with peat and lavender extracts, region unspecified; lavender footbath; oxalis ointment compress to the abdomen, 4× weekly sessions	Observation, schematic progress documentation	Warming sensation, increased attention, improved sleep, relaxation, more able to cope with life’s issues, feeling grounded and more integrated
Deckers et al. (63)	Retrospective case report	61/male, episode of prolonged postoperative ileus, pain	RM with melissa oil to the abdomen; abdominal compress with thuja and argentum ointment, 1×	Observation, schematic progress documentation	Gradual improvement of symptoms over the next 10 days; no prokinetic medications were needed to manage the episode, decrease in pain

Abstract, only abstract of study available; AE, adverse effect; gray, study from gray literature; RM, rhythmical massage; SF-36, Health-Related Quality-of-Life questionnaire short form; PM, Pressel stream massage.

**TABLE 9:** Summary of case reports on compresses.

Reference	Type of study	Participant: Age/gender, diagnosis	Intervention: Modality, substance, body region, number/time span	Main outcome assessment	Main results: Improvements of outcome parameters
Glaser et al. (98) (gray)	Retrospective case series	49/female, recurrent influenza, herpes corneae	Ginger kidney compress, unspecified number	Observation, interviews	Warming sensation, feeling overwhelmed by inner images with following positive processing and feeling of dissolution
		55/female, food intolerance, diarrhea	Ginger kidney compress, 31× in 2 cycles	Observation, interviews	Warming sensation, feeling of release, improved sleep quality, relaxation, feeling of inner support; AE: Tiredness after first treatment, reduction of ginger powder
		50/male, asthma bronchiale	Ginger kidney compress, 9×	Observation, interviews	Warming sensation, relaxation; AE: emerging of problematic thoughts during one treatment
		33/female, asthma bronchiale	Ginger thorax compress, 7×; ginger kidney compresses, number unspecified	Observation, interviews	First AE: Feeling tightness, coughing, after break: warming sensation, improvement of expectoration
		70/female, spastic bronchitis	Ginger thorax compress, number unspecified	Observation, interviews	Warming sensation
		55/female, depression, restlessness	Ginger kidney compress, 7×	Observation, interviews	Warming sensation, relaxation, increased duration of sleep
		48/female, back and joint pain, adipositas	Ginger kidney compress, 10×	Observation, interviews	Warming sensation, relaxation; AE: No pain release
		47/male, back pain, subfebrile temperature, leukocytosis	Ginger kidney compress, 8–10×	Observation, interviews	Warming sensation, but no pain release, relaxation
		68/female, pancreatic carcinoma, abdomen/back pain	Ginger kidney compress, 17×	Observation, interviews	Warming sensation, pain release (only before noon)
		79/female, abdomen carcinoma, skin metastases, ascites	Ginger kidney compress, 12×	Observation, interviews	Warming effect, calming effect on respiration, increased appetite, decreased ascites
		33/male, colon carcinoma, liver metastases etc.	Ginger kidney compresses over 3 weeks, number unspecified	Observation, interviews	First AE: Feeling tightness and restlessness, then warming sensation and relaxation
		48/female, pneumonia	Ginger thorax compress, 1×	Observation, interviews	Increasing warming effect, energized feeling
		83/female, pneumonia after cardiac arrest	Ginger kidney compress, 9×, treatment discontinued	Observation, interviews	First improvement: respiration, expectoration, sleep; later decrease in effect
		56/female, primary chronic polyarthritis, joint pain	Ginger kidney compress, 4×, treatment discontinued	Observation, interviews	First warming sensation, later AE: Weakening, discomfort of the skin in form of burning, itching, and redness
		28/female, melanoma, sleep disturbance	Ginger kidney compress, 2×, treatment discontinued	Observation, interviews	AE: Sensation of wetness and cold, restlessness
		27/female, eating disorder, underweight, sleep disturbance	Ginger kidney compress, 5×, treatment discontinued	Observation, interviews	AE: Subjective sensation of cooling, no feeling of release, continued sleep disturbance
Therkleson (76)	Prospective case study	>65/male, osteoarthritis, pain	Ginger kidney compress, 7× in 7 consecutive days; patch self-treatment at home for a further 24 weeks	Arthritis HAQ, pain VAS, diary; assessment daily, 8 days BT until 6 days AT, and after 24 weeks	Diary: Warming sensation, increase in flexibility and mobility, decrease in pain; improvement of global effect, fatigue, and mobility, continued improvements in global effect, fatigue, and mobility over the 24-week of self-treatment
Schier and Bruchner (86) (gray)	Prospective case study	40/male, seminoma, in the third chemotherapy cycle	Yarrow liver compress during chemotherapy, 5× daily	Observation, interviews, schematic progress documentation, self-reports	Symptom relief compared to the first two cycles of chemotherapy: No symptoms of nausea, loss of appetite or tension during the application days, enhanced appetite, warming sensation, feeling of relaxation
Deckers (89) (gray)	Retrospective case report	20/male, pneumonia, pain	Mustard compress on the chest followed by lavender oil, 1×	Observation, interviews, schematic progress documentation	Hyperemia of the skin followed by pain reduction, deepened breathing, expectoration

AE, adverse effects; AT, after treatment; BT, before treatment; gray, study from gray literature; HAQ, Health Assessment Questionnaire; VAS, Visual Analog Scale.

#### Setting and participant characteristics

Of the 45 included studies, 21 studies originated from Germany (59, 60, 63–65, 69–73, 78, 79, 81, 83, 84, 86, 89, 96, 98, 99, 103), 15 studies originated from Switzerland (66–68, 80, 82, 85, 87, 88, 90, 93, 95, 101, 102), 5 studies originated from New Zealand (61, 62, 74–76), 2 studies originated from the United Kingdom (91, 92), 1 study originated from Brazil (94), and 1 study originated from the United States (100).

Participants were recruited during inpatient treatment in integrative AM clinics in 10 studies (63, 68, 76, 86, 89, 91, 92, 96, 98, 99), during outpatient treatment in integrative AM clinics in 3 studies (64, 66, 67), during inpatient treatment in conventional or unspecified clinics in 7 studies (61, 62, 72, 74, 75, 94, 100), in private practices in 2 studies (78, 81), mainly in primary care practice in 1 study (65), through physician referral or advertisement in 2 studies (59, 73), and through advertisement at a university hospital in 3 studies (69–71). In 17 studies, the recruitment of participants was not specified. All EAAMs to patients were applied complementary to their respective usual treatment regimen if there was one.

In total, 45 studies reported results on 815 participants. Seventy of them participated in different study parts and are, therefore, part of multiple publications. The reported age of the participants ranged from 6 to 83 years, with some studies reporting mean age, some median age, some age ranges, and some not reporting the age of the participants at all. Of the participants in all studies, 74% were female participants and 20% were male participants. In 6% (3 studies), the authors did not report the gender of the participants (60, 74, 94).

Seven studies included patients with various symptoms among the participants (61, 66–68, 83, 94, 98). One study included patients with various chronic diseases, e.g., musculoskeletal or mental diseases (65), and one study included patients with chronic pain (64). Four studies included patients with cancer, e.g., with nausea and fatigue (72, 86, 96, 99). Three studies were on patients with gynecological issues, e.g., with dysmenorrhea or amenorrhea (59, 73, 81). Five studies included patients with neurological disorders, such as hypersensitivity to sensory stimuli after brain injury (91), migraines (103), Morbus Parkinson (88), pain and spasticity after artery stroke (92), and sensory neuropathy (80). Seven studies included patients with orthopedic diseases, of these, 3 studies were on patients with chronic osteoarthritis (62, 74, 76), 2 on patients with spinal disorders (79, 102), 1 on a patient after surgery on humerus fracture (97), and 1 on a pediatric patient with spastic tetraparesis (87). Three studies included patients with pulmonary diseases, such as pneumonia (89), asthma bronchiale (77), and sleep apnea (82). Eight studies included patients with psychiatric or psychological symptoms, out of these, there were 2 studies on patients with burnout syndrome (90, 101); the other was on a geriatric patient with sleep disturbance (84) and on patients with depression and concurrent abdominal complaints (85), exam anxiety (95), psychological trauma (75); 1 included a pediatric patient with restlessness (78) and 1 included a pediatric patient on the autism spectrum (93). One study included patients with acute fever (100) and 1 study included a patient with prolonged postoperative ileus (gastroenterological) (63). Three studies were on healthy adults (69–71), and 1 study performed a qualitative analysis of interviews with nursing experts (60).

#### Intervention characteristics

Of the 45 studies, 34 were on massage interventions and 11 on compress interventions. Table 10 describes the distribution of studies per application type and substance.

**TABLE 10:** The number of studies per application type and substance.

External application/Number of studies *Of which with Substance/Number of studies*
Massages: 34
Rhythmical massage: 23
*Oil with lavender and peat: 2*
*Unspecified/various substances: 21*
Rhythmical embrocation: 6
*Oil with lavender and peat: 2*
*Unspecified/various substances: 4*
Pressel stream massage: 2
*Unspecified/various substances: 2*
Massage with other applications: 3
*Unspecified/various substances: 3*
Compresses: 11
*Ginger powder: 5*
*Yarrow infusion: 5*
*Mustard flour: 1*
*Lemon aroma oil: 1*
*Ginger/mustard/wet/dry: 1*
*Unspecified/various substances: 1*

Of the 34 massage studies, 23 studies were on RM, of which 2 studies were on RM with aroma oil with peat and lavender extracts (69, 70), and 21 studies were on RM with unspecified or various substances (59, 65–67, 73, 77–79, 82, 87, 88, 90–93, 96, 97, 99, 101–103). Six studies were on RE of which 2 were on RE with aroma oil with peat and lavender extracts (64, 84) and 4 were on RE with unspecified substances (60, 80, 85, 95). Two studies were on PM with unspecified substances (81, 83). Three studies were on massages mixed with compresses and/or footbaths: 1 using various aroma oils and ointments (63), 1 using aroma oil and ointment with peat, lavender, and oxalis extracts (75), and 1 using unspecified substances (94).

Of the 11 studies on compresses, 5 studies were on ginger powder compresses (61, 62, 74, 76, 98), 2 on yarrow infusion compresses (72, 86), 1 on a mustard flour compress (89), 1 on lemon aroma oil compresses (100), and 2 on differing compresses, 1 comparing ginger and mustard with neutral dry and wet compresses (71), and 1 describing effects of various compresses (68).

Of the 4 RCTs, 2 compared RM with aroma oil with peat and lavender extracts to RM with neutral oil and sham massage with neutral oil (69, 70), 1 compared RM with heart rate variability biofeedback and treatment as usual (73), and 1 compared yarrow liver compress therapy to treatment as usual (72). One study compared 4 compresses (mustard, ginger, hot water, and hot dry) with different substances within one treatment group (71), and 1 study used different forms of application for ginger compresses (manual application vs. patch) (62). The other studies did not use comparisons or control groups.

### Outcome measures

The 13 included quantitative studies focused on both the subjective assessment of physical outcomes such as symptom manifestation and therapy effectiveness and objectively measured physical and psychological outcomes.

#### Physical measures

Physiological outcomes were assessed using subjective instruments by the 24-Item List of Somatic Complaints (B-L) (70, 71), by measuring the manifestation of disease and symptoms by Visual Analog Scales (70, 72), disease scores and symptom scores (65, 67), and a questionnaire for the state of health assessment (68). Health assessment in patients with osteoarthritis was conducted by Modified Health Assessment Questionnaires for arthritis (MHAQ, HAQ-II) (62), and fatigue in a patient with cancer was assessed using the multidimensional fatigue inventory (MFI-20) (72). Pain intensity was assessed by a numeric rating scale in patients with dysmenorrhea (73), and sensory and affective pain in patients with chronic pain were assessed by the McGill Pain Questionnaire (MPQ), while pain intensity was assessed with a Visual Analog Scale (64). The intensity of headaches during fever was assessed using a rating scale for subjective assessment by the patients (100). The use of analgesics and other medication was assessed in 3 studies (62, 64, 73). Overall therapy effectiveness was assessed in 3 studies using rating scales (62, 65) and the Goal Attainment Scale (67).

Objective measures of physiological parameters assessed autonomic regulation using heart rate variability data from electrocardiogram assessments in 3 studies (66, 69, 73). Body surface temperature was assessed by infrared imaging of the dorsal region in 1 study (66). Body temperature by a clinical thermometer and also pulse, respiratory rate, and blood pressure were assessed in patients with fever (100). One study assessed salivary cortisol (70).

#### Psychological assessments

The mental state was assessed in 3 studies by the Multidimensional Questionnaire on Mental State (MDBF) (70, 71) and Zerssens Adjective Mood Scale (Bf-S) (64, 70). One study used the Diagnostic and Statistical Manual of Mental Disorders (DSM-IV) to assess symptoms of depression (94) and 1 study used the German version of the distress thermometer of the National Comprehensive Cancer Network to assess psychological distress (72). As outcome assessments for the cognitive state, the Mini-mental State Exam (MMSE) and Figures Tests (FT) were used (94). Rating scales were used to assess the experience of relaxation and warmth (71) and additional open questions were used to assess the remarks of participants (70).

#### Physical–psychological assessment

Quality of life (QoL) was assessed by Health-Related Quality-of-Life questionnaire short forms SF-36 (65, 67) and SF-12 (73), World Health Organization Quality of Life questionnaire short form (WHOQOL-bref) (94), and the Quality of life questionnaire of the European Organization for Research and Treatment of Cancer (QLQ-C30) (72).

#### Assessment of long-term effects

All quantitative studies assessed short-term effects, while 3 studies also assessed long-term effects (62, 65, 67).

#### Outcome assessment in qualitative studies and case reports

The qualitative studies included interviews (59–61, 71, 74). The case reports mainly used observations and interviews for outcome assessment, while some case studies additionally used questionnaires for outcome assessment, such as the Health-Related Quality-of-Life questionnaire short form (SF-36) (82, 95, 99), the Arthritis Health Assessment Questionnaire (HAQ) and a Visual Analog Scale for pain assessment (76), and diaries (76, 80, 88, 99) for progress documentation. One case series exclusively used patient self-reports in 2 cases (96).

#### Assessment of adverse effects and safety

While most studies assessed positive effects on the patients, there were 4 quantitative studies (62, 64, 65, 67) and the mixed-methods study (71) which additionally assessed adverse effects. Three case series and reports reported adverse effects (81, 93, 98).

### Outcomes

#### Effect themes

The *Thematic Analysis* of the 45 studies resulted in 4 themes describing different areas of effects (1) physical effects, (2) psychological effects, (3) effect development over time, and (4) adverse effects. The 4 themes contained 7 subthemes. An overview of the themes and subthemes is shown in Table 11, where study clusters are marked when a theme was extracted from a study cluster at least once. Each of the developed themes could be extracted from the outcomes of the massage and compress studies with different characteristics and in some studies with significant effects, as described below.

**TABLE 11:** Effect themes and subthemes.

	Studies on massages				Studies on compresses				
	Rhythmical massage (23 studies)	Rhythmical embrocation (6 studies)	Pressel stream massage	Massage/compress/footbath (3 studies)	Ginger (5 studies)	Yarrow (2 studies)	Mustard (1 study)	Lemon (1 study)	Mixed/different compresses (2 studies)
**1 Physiological effects**									
**1.1 Reactions of the body**									
1.1.1 Decrease in blood pressure	×								
1.1.2 Deepened breathing	×	×			×				×
1.1.3 Improvement of skin texture			×						
1.1.4 Increase of appetite					×	×			
1.1.5 Increase of body temperature/blood circulation	×				×		×		
1.1.6 Increase of libido	×								
1.1.7 Stimulation of HRV	×								
1.1.8 Strengthening/vitalizing/overall improvement	×			×	×	×			
1.1.9 Warming sensation	×	×	×	×	×	×			×
1.1.9.1 Different quality of warmth in ginger/mustard									×
**1.2 Symptom relief**									
1.2.1 General symptom relief/rating as effective	×								×
1.2.2 Expectoration					×				
1.2.3 Discontinuation of medication (pain, sedatives etc.)	×			×	×				
1.2.4 Improvement of ascites					×				
1.2.5 Improvement of asthma symptoms	×								
1.2.6 Improvement of digestion/bloating		×	×	×					
1.2.7 Improvement of fatigue					×	×			
1.2.8 Improvement of fever								×	
1.2.9 Improvement of cognitive functions				×					
1.2.10 Improvement of mobility	×		×		×		×		
1.2.11 Improvement of sensitivity in the legs		×							
1.2.12 Improvement of vertigo	×								
1.2.13 Reduction of headaches		×	×					×	
1.2.14 Reduction of pain and muscle relaxation (local)	×	×	×	×	×		×		
1.2.15 Regulation of menstruation			×						
**2 Psychological effects**									
**2.1 Activating effects**									
2.1.1 Feeling of release, liberating	×	×	×		×				
2.1.2 Improvement of confidence		×							
2.1.3 Improvement of mood/feeling light	×	×	×	×		×			×
2.1.4 Improvement of quality of life/health satisfaction	×			×	×				
2.1.5 Psychological activation						×			
**2.2 Relaxing effects**									
2.2.1 Feeling of relaxation	×	×		×	×	×			×
2.2.1.1 Relaxation only in the after rest		×							
2.2.2 Improvement of sleep	×	×	×	×	×				
2.2.3 Pleasant and restful feeling	×	×				×			×
**2.3 Improvement of competencies**									
2.3.1 Feeling in balance/stable/Sense of coherence	×	×	×	×	×				×
2.3.2 Improved regulation of anxiety	×	×							
2.3.3 Improvement in symptom management (pain etc.)	×				×	×			
2.3.4 Improvement of competencies in daily life	×	×	×		×				
2.3.5 Improvement of self-awareness	×	×		×	×				
2.3.6 Improvement of social skills	×				×				
**3 Development of effects over time**									
3.1 Being uncaged à re-identifying à being empowered		×							
3.2 Long-term effect	×	×							×
3.3 Improvement of effect after multiple applications	×	×	×	×	×				
3.4 Decrease in effect after multiple applications					×				
**4 Adverse effects**									
**4.1 Physiological adverse effects**									
4.1.1 No symptom relief/no effect		×			×				×
4.1.2 Symptom aggravation	×								
4.1.3 Exhaustion/tiredness			×		×				
4.1.4 Flattening of breath		×							
4.1.5 Irritation of the skin					×				
4.1.6 Occurrence of cardiac palpitations	×								
4.1.7 Occurrence of coughing					×				
4.1.8 Occurrence of hypotension	×								
4.1.9 Occurrence of pain: Abdomen			×						
4.1.10 Occurrence of pain: Headache, dental pain			×						
4.1.12 Occurrence of vertigo	×								
4.1.13 Sensation of cold and wetness					×				
**4.2 Psychological adverse effects**									
4.2.1 Emotional agitation/feeling depressed/restless	×		×		×				
4.2.2 Overwhelmed by touch	×								

#### Physical effects of massages

In the studies on massage interventions, physiological effects were presented in the form of various reactions of the body and symptom relief. Reactions of the body showed in form of improvement of general condition and vitalization (59, 63, 65, 67, 82, 83, 90, 96, 99, 102), a warming sensation in the whole body (75, 80, 81, 84, 85, 87, 95, 96, 101), deepened breathing (84, 87, 88, 93, 95, 96, 102), an increase in body temperature and blood circulation (66, 87, 93, 97, 101, 102), a decrease in blood pressure (77), a stimulation of heart rate variability (HRV) (66, 69), an improvement of skin texture (81) and an increase in libido (82). Symptom relief after massage interventions was shown in the form of general symptom relief and general rating of applications as effective (65, 67), a reduction in local pain and muscle relaxation (63, 64, 73, 81, 83, 97, 99, 101, 102), an improvement of mobility (83, 87, 88, 96, 97), improvements of digestion and bloating (63, 81, 85), reductions in headaches (81, 95), improvements in asthma symptoms (77), an improvement of sensitivity in the legs (80), an improvement of vertigo (82), a regulation of the menstrual cycle (81), improvements in cognitive function (94), and the possibility for discontinuation of analgesia and sedatives (63, 77).

#### Significant effects of massages on physical outcomes

The quantitative studies on massage interventions revealed significant effects for some of the stated physiological outcomes: RM treatment for 3 months compared to treatment as usual (TAU) led to a significant reduction in pain intensity in patients with dysmenorrhea between groups (*p* < 0.01) (73). RM led to a significant pre-post reduction in disease manifestation (short-term: *p* < 0.001/long-term: *p* < 0.001) and symptom scores (short-term and long-term: *p* < 0.001) in patients with various indications (67) and to a significant reduction in disease manifestation (*p* < 0.001) and symptom scores (*p* < 0.001) in patients with chronic disease (65). After RM, the patients’ rating of therapy goal attainment was also significantly higher (short-term and long-term: *p* < 0.001) in the various indications sample (67). RE had significant positive effects on sensory (*p* < 0.001, *d* = 0.55) and affective (*p* < 0.001, *d* = 0.85) pain, and pain intensity (*p* < 0.001), in patients with chronic pain (64). An intervention using embrocations together with compresses and footbaths led to a significant increase in cognitive function (*p* = 0.008) and incidental (*p* = 0.003) and immediate (*p* = 0.006) memory as well as an increase in evocation capacity (*p* = 0.001) in elderly patients (94). Significant positive effects in autonomic regulation (assessed by HRV) between treatment with RM with aroma oil over a treatment with RM with neutral oil (*p* < 0.01 in most HRV parameters) and over a treatment with sham massage (*p* < 0.01 in most HRV parameters) were evident in healthy adults (69). RM also had significant positive effects on autonomic regulation (HRV) (*p* < 0.001 in most HRV parameters) and surface temperature (IRI) (*p* < 0.001) in patients with various indications (66).

#### Physical effects of compresses

Effects on physical parameters in the studies on compress interventions were extracted as reactions of the body such as an improvement of general condition and vitalization (61, 62, 74, 98), warming sensations in the whole body (61, 71, 74, 76, 98) that were described as a different quality of warmth in ginger and mustard (71), deepened breathing (71, 98), an increase in appetite (86, 98), and increases in body temperature and blood circulation (61, 76, 89, 98, 100). Compresses led to symptom relief in the form of general symptom relief and rating of treatments as effective (68), improvements in fatigue symptoms (62, 72), improvements in mobility (62, 74, 76, 89), reduction in local pain and muscle relaxation (62, 74, 76, 89, 98), a reduction in headaches (100), a stimulation of expectoration (98), and improvements of fever (100) and ascites (98).

#### Significant effects of compresses on physical outcomes

Significant effects of compress interventions on physical outcomes were significantly reduced fever (measured temperature) (after treatment: *p* = 0.027, after 60 min: *p* = 0.006) and headaches (*p* = 0.01) after lemon compresses (100). After ginger compresses, significant effects on healthy adults were deepened breathing (*p* = 0.03), slowed breathing (*p* < 0.001), and the sensation of warmer hands (*p* = 0.03) and warmer feet (*p* < 0.001) (71).

#### Psychological effects of massages

The effects of massages on psychological outcomes were presented as activating effects such as an improved mood and feeling light (64, 67, 70, 80–82, 87, 94–97, 99, 102), a feeling of release and liberation (60, 81, 87, 95, 96), improvements in QoL and health satisfaction (73, 82, 94, 99, 101), and improved confidence (95). The relaxing effects of massages were improvements in sleep (75, 78, 80–82, 84, 88, 90, 99), a pleasant restful feeling (59, 65, 78, 84, 85, 90, 99), and a feeling of relaxation (59, 70, 75, 80, 84, 87, 88, 90, 95, 96, 99). Some participants described the effects of improved competencies through massages, like strengthening through an increased perception of balance and meaning (60, 75, 80, 81, 83, 85, 95, 96, 99), an improved self-awareness (59, 70, 75, 79, 84, 87, 88, 90, 95, 96, 99, 103), an improvement of competency in daily life (59, 60, 79, 81, 82, 87, 88, 93, 95, 102, 103), an improved ability to regulate anxiety (82, 91, 95, 102), improved capacities for symptom management (79, 91, 92, 97), and improved social skills (78, 82, 90).

#### Significant effects of massages on psychological outcomes

Rhythmical massage had significant positive effects on QoL in patients with chronic diseases (physical and mental: *p* < 0.001) (65), dysmenorrhea (no *p*-value reported, *d* = 0.60) (73) and in patients with various indications (physical short-term: *p* = 0.005/physical long-term: *p* = 0.005, mental short-term: *p* < 0.001/mental long-term: *p* < 0.001) (67). RM significantly improved mood (*p* < 0.001, *d* = 0.81) in patients with chronic pain (64). An intervention using embrocations along with compresses and footbaths led to a significant increase in QoL (Physical: *p* = 0.05, psychological: *p* = 0.007, social: *p* = 0.048, environment: *p* = 0.02), and a significant decrease in depression (*p* = 0.002) in elderly patients (94).

#### Psychological effects of compresses

The effects of compresses on psychological outcomes were extracted in the form of activating, relaxing effects, and effects of competencies as well: After compresses, patients described improvement in mood and feeling light (71, 86), feelings of release and liberation (98), improvements in QoL and health satisfaction (62), and a feeling of psychological activation (72). Feelings of overall relaxation (71, 72, 74, 98), pleasant and restful feelings (68, 86), and an improvement of sleep (98) were described after compressing as well. Furthermore, strengthening through an increased perception of balance and meaning (71, 76), improvements in symptom management (76, 86), daily life competencies (61, 74), self-awareness (61), and social skills (74) also showed after compress interventions.

#### Significant effects of compresses on psychological outcomes

Daily yarrow liver compresses for 7–14 days led to a significant reduction in fatigue (*p* = 0.015), tension (*p* = 0.044), lack of drive (*p* = 0.028), and an improvement of the scale on reduced motivation in fatigue (*p* = 0.035) compared to TAU in patients with cancer during radiation therapy (72). After ginger compresses, healthy adults were significantly more relaxed (*p* < 0.001) and emotionally balanced (*p* = 0.03) (71). Their mood was significantly improved after dry compresses and compresses using hot water and ginger (all compresses: *p* < 0.05), and calmness was significantly improved after dry, hot water, ginger, and mustard compresses (all compresses: *p* < 0.05) (71).

#### Development of effects in massage

In the massage studies, development of effects over time was described in manifesting an increase of effects after multiple applications in various studies (63, 82–84, 96, 99) as well as long-term effects (65–67, 85, 88, 103), and a pattern of effect consisting of a feeling of liberation, followed by a feeling of re-identification and a feeling of empowerment (60).

#### Significant long-time effects of massages

One study on patients with various indications reported significant long-term effects in a pre-post reduction in disease manifestation (*p* < 0.001), symptom scores (*p* < 0.001), therapy goal attainment (*p* < 0.001), and QoL (physical: *p* = 0.005, mental: *p* < 0.001) (67).

#### Development of effects in compresses

The studies on compresses depicted a development of effects over time as well, shown in an increase of effects after multiple applications (62, 76) as well as a decrease of effect over time in some cases (98) and long-term effects (68). No significant long-term effects of compresses were reported.

#### Adverse effects of massages

Adverse physical effects in massage studies were symptom aggravation, arterial hypotension, cardiac palpitations, vertigo after treatment (65), exhaustion and tiredness after the treatment (81), flattening of breath (85), pain in the abdomen (81), headaches, and dental pain (65, 81, 103). The adverse psychological effect after massages was emotional agitation and a depressed and restless feeling (59, 81), and being overwhelmed by the interpersonal touch (93).

#### Adverse effects of compresses

In the compress studies, adverse effects were only reported in a case series on ginger compressions in the form of exhaustion after the treatment, irritation of the skin, cough, an unpleasant sensation of cold and wetness, emotional agitation, and feelings of restlessness (98).

### Study quality

Since the study methodologies, the outcome measurements and the samples were heterogeneous and the studies were generally of low quality, the data did not allow for meta-analysis of the results. Study quality according to LoE, QA-Tool, and RoB is presented in Table 12.

**TABLE 12:** Summary of the assessment of study quality and risk of bias in the quantitative studies.

			Quality assessment							Risk of bias assessment								
	Reference	Level of evidence	Selection bias: Representation of population	Study design: Design, randomization	Confounders: Detection and controlling of confounders	Blinding of outcome assessors and participants	Data collection methods: Tools shown to be valid + representative	Withdrawals and dropouts: Reporting of dropouts, completing	Quality: Global rating	Random sequence generation: Selection bias	Allocation concealment: Selection bias	Blinding of participants and personnel: Performance bias	Blinding of objective outcome assessment: Detection bias	Blinding of subjective outcome assessment: Detection bias	Incomplete outcome data: Attrition bias	Selective reporting: Reporting bias	Other bias	Risk of bias: Global rating
Massages	Kanitz et al. (70)	II	strong	strong	strong	mod.q.	strong	strong	strong	low	low	high	high	high	low	low	low	mod.r.
	Seifert et al. (69)	II	mod.q.	strong	strong	mod.q.	strong	mod.q.	strong	low	low	high	high	n.a.	low	low	high	mod.r.
	Vagedes et al. (73)	II	weak	strong	mod.q.	weak	strong	strong	weak	low	low	high	high	high	high	low	low	mod.r.
	Hamre et al. (65)	IV	strong	mod.q.	weak	weak	strong	mod.q.	weak	high	high	high	uncl.	high	low	low	uncl.	high
	Wälchli et al. (67)	IV	mod.q.	mod.q.	weak	weak	strong	mod.q.	weak	high	high	high	uncl.	high	low	low	uncl.	high
	Wälchli et al. (66)	IV	mod.q.	mod.q.	weak	weak	mod.q.	mod.q.	weak	high	high	high	high	n.a.	low	uncl.	high	high
	Ostermann et al. (64)	IV	mod.q.	mod.q.	weak	weak	strong	strong	weak	high	high	high	n.a.	high	low	low	uncl.	high
	Vieira et al. (94)	IV	mod.q.	mod.q.	weak	weak	weak	strong	weak	high	high	high	n.a.	high	low	low	high	high
Compresses	Ghadjar et al. (72)	II	mod.q.	strong	strong	weak	strong	weak	weak	low	uncl.	high	n.a.	high	high	low	high	high
	Stritter et al. (71)	IV	weak	mod.q.	mod.q.	mod.q.	strong	strong	mod.q.	high	high	high	n.a.	high	low	low	high	high
	Klich-Heartt (100)	IV	weak	weak	weak	weak	mod.q.	weak	weak	high	high	high	high	high	low	low	high	high
	Therkleson (62)	IV	weak	mod.q.	weak	weak	strong	strong	weak	high	high	high	n.a.	high	low	high	high	high
	Simoes-Wüst et al. (68)	VI	weak	weak	weak	weak	weak	weak	weak	high	high	high	n.a.	high	low	low	high	high

Strong, strong quality; mod.q., moderate quality; weak, weak quality; low, low risk of bias; mod.r., moderate risk of bias; high, high risk of bias; uncl., unclear risk of bias; n.a., not applicable.

#### Level of evidence

**FIGURE 3:**
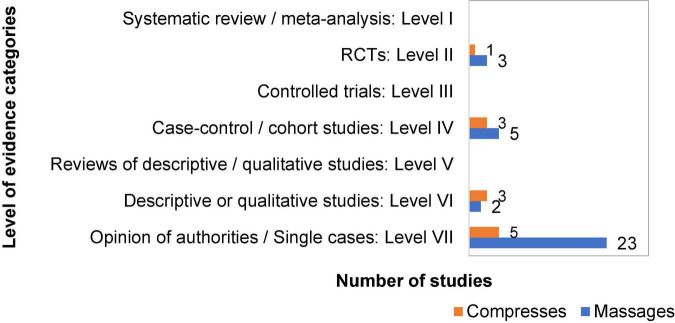
Studies on EAAM per level of evidence category reported in absolute terms.

The assignment of the individual studies to the heuristic of LoE resulted in a distribution of the studies between the levels according to their methodology (Figure 3). Level II was assigned 3 studies on massages (69, 70, 73) and 1 study on compresses (72). Level IV was assigned 5 studies on massages (64–67, 94) and 3 studies on compresses (62, 71, 100). Level VI was assigned 2 studies on massages (59, 60) and 3 studies on compresses (61, 68, 74). Level VII was assigned 23 studies on massages (75, 77–85, 87, 88, 90–93, 95–97, 99, 101–103) and 5 studies on compresses (63, 76, 86, 89, 98). Mixed methods studies were counted as quantitative studies (Level IV). Studies exploring a mixed intervention of massages and other application types were counted as massage studies.

#### Study quality assessment

Out of the 13 quantitative studies, 2 had strong quality (69, 70), 1 had moderate quality (71), and 10 had weak quality (62, 64–68, 72, 73, 94, 100) (Table 12). Two studies showed strong quality in representation of the population (selection bias), while 6 studies had moderate and 5 had weak quality in this domain. The study design was of strong quality in 4 studies (RCTs), moderate in 7 studies, weak in 2 studies, with 3 strong, 2 moderate, and 8 weak in the detection and controlling of confounders. Blinding of outcome assessors and participants was moderate in 3 studies and weak in 10 studies. Data collection methods were strong in 9 studies, moderate in 2 studies, and weak in 2 studies. Reporting of withdrawals and dropouts was strong in 6 studies, moderate in 4 studies, and weak in 3 studies.

#### Risk of bias assessment

Out of the 13 quantitative studies, 3 had a moderate risk of bias (69, 70, 73) and 10 had a high risk of bias (62, 64–68, 71, 72, 94, 100) (Table 12). The risk of selection bias was high in most studies as only 4 studies reported random sequence generation and only 3 studies reported adequate allocation concealment. While no study reported blinding of participants or personnel to the intervention, the study comparing different compresses reported blinding of participants to the used substances (71). No study reported adequate blinding of outcome assessors. Attrition bias was low in most studies as well as reporting bias. Other bias was caused mainly by small sample sizes and was only low in two studies.

## Discussion

This mixed methods systematic review of 45 studies on EAAM included 34 studies on massage interventions and 11 studies on compress interventions. In both groups, various substances were applied. The methodologies of the included studies ranged from RCTs to cohort, retrospective, mixed methods and qualitative studies, and case series and reports. While a broad range of possible indications for applications could be identified by the *Thematic Analyses* of all included studies, limited statistically significant evidence for improvements was found in the outcomes. The safety of the different EAAMs needs further detailed evaluation.

### External and internal validity

Overall, the results of this review are applicable mainly to European patients interested in integrative medical treatments and might be most applicable to the female gender. Further studies on EAAM applied to different participants are needed to ensure the external validity of the results. The employed methodology and the results of the quality and risk of bias assessments limit the interpretability of the results. More studies of high quality with low risk of bias need to be conducted and compared before conclusions can be drawn about the impact of EAAM.

### Touching body, soul, and spirit: Intersections with other theories on health

The results on the effects of EAAM in this review suggest various health promoting effects, and therefore, match the findings on the health promoting capacities of the application of warmth and substances (14, 16–18, 21–24) as well as on touch and massage (9, 30, 31). The importance given to rhythmical stroking in massages matches the findings on the impact of a certain pace and pressure to stimulate especially C-tactile afferents in the skin to reach a positive effect and affective value (29).

While scientific medical and psychological research investigates if and how treatments affect individuals, Anthroposophy and AM already present views on effect mechanisms (104, 105). Suiting interpretations of the effect mechanisms of EAAM could also be detected in some of the included studies of this review, postulating a stimulation of self-regulation by the integration of the *Four Levels of Formative Forces* and/or through the regulation of the *Threefold Functional Subsystems* of the organism (80, 83–89, 95, 96, 99–101). In addition, the emergent effect themes could be interpreted as the influence of EAAM on the *Formative Forces* of the body (*Material Body* and vital *Etheric Body*), soul, and spirit according to AM (34): Physiological effects could be translated as affecting the body (*Material Body* and vital *Etheric Body*), psychological effects as affecting the soul, and the effects on psychological competencies could be translated as affecting the spirit.

However, as underlined by the findings of this review, the explanations of EAAM effect mechanisms according to AM do not translate directly into evidence according to conventional scientific research standards. To bridge this gap between the findings of this review and conventional scientific research, a digression into other scientific theories on health can provide alternative models to explain the effects of EAAM.

#### Benefits of interpersonal attention

External applications from anthroposophic medicine use interpersonal attention and touch. While the health-promoting capacities of touch have been described repeatedly (30, 106), two theories from psychological research specifically emphasize the importance of social contact to promote health. The *Social Baseline Theory* states that through social regulation of emotion, interaction with other people helps individuals to conserve somatic and neural resources, inhibit the release of stress hormones, reduce the risk of developing physical and mental illnesses, and promote health and longevity (107). The *Tend and Befriend Model* defines interpersonal tending as a natural reaction to stress and therefore as a baseline condition for health (108). These findings support the assumption that EAAM, as applications involving interpersonal contact and attention, may support health. In addition, findings suggest advantages for the practitioners when giving touch as well (109).

#### Impact of intentions and expectations

External applications from anthroposophic medicine are performed in a defined recurrent way, similar to rituals, and are often based on beliefs in their specific effect mechanisms according to AM. An approach to explain the efficacy of including a certain meaning in medical treatments is the *Meaning Model*, proposing that positive responses to applications may result from patients feeling listened to and attended to by caregivers, receiving an explanation for their disease that is consistent with their own worldview, receiving care and compassion, and experiencing an increased sense of mastery or control over their health (110). The setting of EAAM may, therefore, promote placebo effects (111) that can be used to ensure maximum benefit for patients (112, 113).

#### Holistic, positive, and dynamic approach to health

External applications from anthroposophic medicine are presumed to have health-promoting effects, to affect health holistically and dynamically. Holistic and positive support models underscore this, such as approaches of positive psychology and positive health [Seligman (114)] and person-centered medicine (115) as well as the *Health Wellness Model*, which promotes the integration of body, mind, and spirit to promote health (116). The consideration of spiritual factors for health promotion is mentioned in the literature as well, proposing that health care that addresses spiritual needs may contribute to recovery (117). The salutogenic definition of health defines a strong *Sense of Coherence* where one perceives the world as comprehensible, manageable, and meaningful as an indicator of health (38). Dynamic well-being conditioned by biopsychosocial potential (118) and health-defining heterostasis in the body (38, 39) are other concepts similar to the concept of flexible regeneration in organisms such as the EAAM underlying intention to stimulate self-regulation of the organism through treatments in order to enable healing (40, 41).

### Strengths and limitations of this review

This is the first review of EAAM. We used the design of a mixed methods systematic review to cover the heterogeneous empirical literature on the different types of EAAM, sample characteristics, methodologies, and outcome parameters (52). The broad scope of the review and the extensive literature search process enables a comprehensive overview of the areas of use and effectiveness of EAAM. Studies in English and German were included, covering most of the available literature on the topic. The quality of the included studies and the applicability of the results were assessed. In order to sort the heterogeneous result data of the included studies, we decided to use the approach of a *Thematic Analysis*. While using this method to summarize results is not common in review papers, it was quite helpful to inductively get an overview of the effect patterns in the included studies.

However, the heterogeneity of the included studies limits their generalizability. Only a small number of quantitative studies could be identified, which limits the expressiveness of the review. Many of the studies were only accessible in the form of gray literature and/or abstracts. As assessed by the LoE classification, the validity of the included studies had to be described as low in many of the studies. Study quality was assessed as low and the risk of bias as high in most studies. The safety of EAAM could only be assessed qualitatively on the basis of a few of the included studies reporting it.

We used a combination of the 3 tools LoE assessment, QA-Tool, and RoB to sort and adequately evaluate the quality and validity of the heterogeneous studies. Quality assessment of the included studies was conducted by two members of the research group (IM, JE). However, the QA-Tool and the RoB are repetitive in some domains and would have benefited from better tailored tools for the evaluation.

### Implications for research and clinical practice

The implementation of complementary EAAM in clinical practice can be an opportunity to consider the patient’s needs for caregiving as well as spiritual needs in some patients in a manner of person-centered medicine.

Since the interpretability of the evidence found in this review is limited by the methodological quality of the included studies, we endorse studies of high quality on the different EAAM for different patient groups. These studies should ensure rigorous methodology and reports (119). In order to gain a deeper understanding of the interpretations of the effect mechanisms of EAAM according to Anthroposophy, a phenomenological analysis (120) based on the result and discussion sections of the included studies and/or theoretical manuscripts on EAAM might be of merit.

Users should adhere to the procedure specifications of the different EAAMs, and caution is advised with regard to the mentioned adverse effects. EAAM is not indicated in patients with adverse attitudes toward touch or in patients with sensitive skin or tissue damage. As illness was described in sections by Steiner and Wegman as depending on personal faculties (37), the underlying views of AM might be perceived as discriminating against people with diseases. When applying EAAM, users should, therefore, be cautious about their intentions with the applications. Furthermore, it is strongly advised to use EAAM complementarily to evidence-based treatments to promote well-being as intended (33, 121).

## Conclusion

The mixed methods systematic review illustrates the potential benefits of the different EAAM modalities as complementary treatments. The data reveal a broad spectrum of effect themes, suggesting that EAAM is suitable to address physical and psychological health indicators by improving the general condition and inducing symptom relief as well as psychologically activating and relaxing effects. Limitations in study quality, varying application modalities, different outcome assessments, and different sample characteristics complicate a substantiated comparison of the outcomes. We recommend further clinical studies exploring the effects and safety of distinguished EAAM modalities on defined patient groups to determine to what extent EAAM can be considered an effective and safe treatment option.

## Data availability statement

The original contributions presented in this study are included in the article/supplementary material, further inquiries can be directed to the corresponding author.

## Author contributions

IM contributed to the conceptualization, methodological planning, data analysis, writing, and revision of the manuscript. SB contributed to the revision and editing. JE contributed to the data analysis. HC and GS contributed to the supervision, revision, and editing. WS contributed to the conceptualization, supervision, revision, and editing. All authors contributed to the article and approved the submitted version.

## Footnotes

1
https://www.anthromedics.org

2
https://www.cambase.de

3
https://www.cam-quest.org

4
https://www.researchgate.net

5
https://www.opengrey.eu/
